# Multiple Omics Analyses Reveal Activation of Nitrogen Metabolism and Flavonoid Glycosylation in *Toxicodendron vernicifluum* Under High Temperature

**DOI:** 10.3390/biology13110876

**Published:** 2024-10-28

**Authors:** Guoqing Bai, Ruiwen Ding, Qizhen Su, Xiaomin Ge, Shasha Li, Huiying Shang, Aiguo Zhao, Chen Chen

**Affiliations:** 1 Shaanxi Engineering Research Centre for Conservation and Utilization of Botanical Resources, Xi’an Botanical Garden of Shaanxi Province, Institute of Botany of Shaanxi Province, Xi’an 710061, China; bgq@ms.xab.ac.cn (G.B.); gxm@xab.ac.cn (X.G.); lishasha@xab.ac.cn (S.L.); shanghuiying@outlook.com (H.S.); 2 East China Academy of Inventory and Planning of NFGA, Hangzhou 310019, China; dingrwhdy@163.com; 3 Qinling National Botanical Garden, Xi’an 710061, China; suqizhen-021@163.com; 4 College of Forestry, Northwest A&F University, Yangling 712100, China

**Keywords:** *Toxicodendron vernicifluum*, high temperature, alkaloid, flavonoid

## Abstract

Lacquer trees (*Toxicodendron vernicifluum*), economically vital, face high-temperature stress in summer. Transcriptomic, proteomic, and metabolomic analyses were employed to investigate the mechanisms by which lacquer trees respond to high temperatures. High-temperature treatment led to notable metabolite changes with 224 upregulated and 69 downregulated. Indole-3-acetic acid remained stable while abscisic acid decreased, with increases in jasmonic acid and jasmonoyl-L-isoleucine indicating complex hormonal responses. JAR1 and ABA 8′-hydroxylase encoding genes were upregulated. The rise in JAs boosted the alkaloid content and activated nitrogen transport. High temperatures also increased specific amino acids and upregulated aminotransferase and protease-encoding genes. Metabolomic analysis showed elevated flavonoid glycosides and the upregulation of glycosyltransferase genes. WPCNA found 35 protein modules involved in secondary metabolite biosynthesis, protein phosphorylation, and signal transduction. Protein–protein interaction analysis revealed MYC6’s link with flavonoid biosynthesis, indicating its role in promoting flavonoids.

## Simple Summary

Lacquer trees are susceptible to high-temperature stress during the summer, which affects their metabolism and management. Research indicates that elevated temperatures enhance the levels of some alkaloids, quinones, and amino acids, signaling active nitrogen metabolism. This stress also led to the degradation of abscisic acid and an increase in the jasmonic acid level. Additionally, restructuring gene expression in lacquer trees triggered metabolic adjustments and promoted the synthesis of certain glycosylated flavonoids. These findings illuminate the metabolic and molecular responses to heat stress, providing valuable insights for enhancing lacquer tree resilience and productivity in a changing climate, thereby benefiting both plant science and agricultural practices.

## 1. Introduction

High-temperature stress poses a significant threat to plant growth, development, and productivity worldwide [1]. As global temperatures continue to rise due to climate change, understanding the mechanisms by which plants respond and adapt to high temperatures is of paramount importance. High-temperature stress can lead to a cascade of physiological, biochemical, and molecular changes in plants, ultimately impacting various cellular processes [2]. At the physiological level, high temperatures can disrupt photosynthesis, leading to reduced carbon assimilation and altered plant metabolism [3]. Heat stress also induces oxidative stress by generating reactive oxygen species (ROS), which can damage cellular components, such as membranes, proteins, and DNA. ROS play a critical role in plant hormone signaling by acting as secondary messengers that modulate responses to stress and development; for instance, they enhance signaling pathways for hormones like abscisic acid during drought stress, trigger ethylene production in response to pathogens, and influence processes such as cell expansion and differentiation [4]. Furthermore, high temperatures can disrupt the water balance in plants, leading to dehydration and impaired nutrient uptake [4]. Biochemically, plants respond to high-temperature stress by synthesizing heat-shock proteins (HSPs), which act as molecular chaperones to refold denatured proteins and maintain cellular homeostasis [5]. Additionally, plants accumulate compatible solutes such as proline and sugars to mitigate the effects of osmotic stress caused by dehydration [6]. At the molecular level, high-temperature stress triggers changes in gene expression patterns, leading to the activation of heat stress-responsive genes and the repression of genes involved in growth and development. Transcription factors such as heat-shock factors (HSFs) and dehydration-responsive element-binding proteins (DREBs) play key roles in regulating the expression of stress-responsive genes [2,7]. High-temperature stress impacts various metabolic pathways and leads to alterations in the composition and abundance of metabolites. At the primary metabolism level, heat stress disrupts photosynthesis, leading to reduced carbon assimilation and altered carbohydrate metabolism [8]. Additionally, high temperatures can perturb nitrogen metabolism, leading to the accumulation of toxic ammonia and impairing nitrogen assimilation processes [9]. Secondary metabolites, including phenolics, flavonoids, terpenoids, and alkaloids, play crucial roles in plant defense mechanisms and adaptation to environmental stresses [10]. High-temperature stress can modulate the biosynthesis and accumulation of secondary metabolites, either increasing or decreasing their levels depending on the plant species, genotype, and stress intensity [3]. Understanding the intricate relationship between metabolites and high-temperature stress is crucial for elucidating plant responses and developing strategies to enhance stress tolerance and resilience.

Lacquer trees (*Toxicodendron vernicifluum*) are renowned for their production of secondary metabolites, which play crucial roles in defense against herbivores, pathogens, and environmental stresses. However, the relationship between lacquer tree secondary metabolites and high-temperature stress is complex and multifaceted. Heat stress disrupts metabolic pathways involved in secondary metabolite biosynthesis, leading to alterations in the profile and abundance of these compounds. For example, phenolic compounds, which contribute to plant defense against oxidative stress, may accumulate in response to high-temperature stress, serving as antioxidants to mitigate oxidative damage [3]. Similarly, flavonoids, terpenoids, and alkaloids, other important classes of secondary metabolites in lacquer trees, may exhibit variable responses to high temperatures. Some compounds within these classes may increase in abundance as part of the plant’s defense mechanisms, while others may undergo degradation or alterations in composition under prolonged heat stress conditions. Integrated omics approaches, including metabolomics, proteomics, and transcriptomics, offer valuable tools for elucidating the regulatory mechanisms underlying these responses. Understanding the intricate relationship between lacquer tree secondary metabolites and high-temperature stress is essential for developing strategies to enhance stress tolerance and resilience in lacquer tree ecosystems. Further research efforts aimed at unraveling the molecular mechanisms governing these interactions will contribute to the sustainable management and utilization of lacquer tree resources in the face of climate change.

## 2. Materials and Methods

### 2.1. Plant Material and Growth Conditions

*T. vernicifluum* seeds (‘Gaobachi’, 2n = 2x = 30) were planted in hole trays filled with seedling substrate with nitrogen level of 200 kg hm^2^. These trays were positioned in a room maintaining a constant temperature of 25 ± 1 °C, with a photoperiod of 14 h, irradiance set at 240 µmol m^−2^ s^−1^, and relative humidity at 80%. Seedlings with four to five main leaves were transferred to a light incubator set at a constant temperature of 45 ± 1 °C, with the same photoperiod, irradiance, and 80% relative humidity. After 1 day of treatment, control and treated samples were collected, washed twice with deionized water, weighed, frozen in liquid nitrogen, and stored at −80 °C. Each treatment was replicated independently three times. The high-temperature-treated seedlings and control seedlings of lacquer tree are shown in Supplementary Figure S1.

### 2.2. Metabolome Profiling

Next, 50 mg of vacuum freeze-drying sample powder was extracted with 1.2 mL of 70% methanolic aqueous extract and vortexed once every 30 min for 30 s, for a total of 6 times. After centrifugation (12,000 rpm, 3 min), the supernatant was aspirated and filtered through a microporous membrane (0.22 μm pore size) and stored in the injection vial for UPLC-MS/MS analysis. The sample extracts were analyzed using an UPLC-ESI-MS/MS system (UPLC, ExionLC™ AD (San Diego, CA, USA), https://sciex.com.cn/ (accessed on 22 October 2024); MS, Applied Biosystems 4500 Q TRAP (San Diego, CA, USA), https://sciex.com.cn/ accessed on 22 October 2024) followed Chen’s protocol [11]. The effluent was alternatively connected to an ESI-triple-quadrupole-linear ion trap (QTRAP)-MS/MS. The metabolites were identified using the compounds database from MetWare (Shanghai, China).

### 2.3. Transcriptome Profiling

Total RNA was extracted using a Plant RNA Extraction Kit (Takara Bio, Beijing, China) and then reverse-transcribed into cDNA. RNA sequencing was performed using the Illumina HiSeq 2500 platform at Metware Biotechnology Co., Ltd. (Wuhan, China). The sequences were assembled by Trinity [12]. Gene function was annotated using DIAMOND through blast Unigene with KEGG, NR, Swiss-Prot, GO, COG/KOG and Trembl. Gene expression analysis was performed using the RSEM package 1.4. DESeq2 was used for differential expression analysis (FDR < 0.05 and |log2 FC (fold change)| ≥ 1). Enrichment of the DEGs was analyzed using the GO and KEGG databases. StringDB (http://string-db.org/ accessed on 22 October 2024) protein–protein interaction (PPI) database was used for protein interaction analysis.

### 2.4. Proteome Profiling

The plant samples were powdered in liquid nitrogen, and proteins were extracted using lysis buffer (7 M urea, 2 M thiourea, 4% SDS, 20 mM Tris-HCl (pH 8.5), 1 mM PMSF, 2 mM EDTA) on ice for 5 min. DTT was then added to a final concentration of 10 mM, followed by sonication in an ice bath for 5–15 min. The lysate was centrifuged at 13,000× *g* and 4 °C for 20 min, and the supernatant was transferred to a new centrifuge tube. Add 4 volumes of cold acetone, incubate at −20 °C for 2 h, centrifuge and discard the supernatant; add 1 mL of cold acetone (containing a final concentration of 10 mM DTT) to the precipitate, vortex, and incubate at −20 °C for 30 min; centrifuge at 13,000× *g*, discard the supernatant, and air dry the protein precipitate. The proteins were resuspended and alkylated in a darkroom, followed by precipitation with acetone. Subsequently, the protein was resuspended in a solution containing 8 M urea/100 mM TEAB (pH 8.0). The protein concentration was measured using the BCA method, and 100 μg of protein from each sample was subjected to trypsin digestion at a mass ratio of 1:50 (trypsin to protein) and allowed to digest overnight at 37 °C. The digested peptides were then desalted using a C18 (Cartridge) cartridge and vacuum freeze-dried. The freeze-dried peptide powder was reconstituted in 0.1% formic acid aqueous solution to a concentration of 0.1 µg/µL and stored at −20 °C for future use. The proteomic determination methods were described by Prianichnikov et al. [13], Metware Biotechnology Co., Ltd. (Wuhan, China). The resulting MS/MS raw data were searched against the Toxicodendron vernicifluum transcriptome database using DIA-NN (v1.8.1) with a 1% false-discovery rate (FDR). The proteins were annotated using Gene Ontology (GO), KOG functional classification, Pfam, KEGG pathways, and signal peptides (SignalP). Proteins with fold change (FC) > 1.5 or FC < 0.6667, and *p*-value < 0.05 are defined as significantly differentially accumulated proteins. A correction for multiple hypothesis testing was carried out using standard FDR control methods, with a corrected *p* ≤ 0.05 considered significant. R(WGCNA) v1.69 was used for protein expression module analysis (mergeCutHeight = 0.25).

### 2.5. Gene Expression via Quantitative Real-Time PCR

The specific primers for 20 unigenes are provided in Supplementary Table S1. Reverse-transcription quantitative PCR (RT-qPCR) was performed using a CFX96 Real-Time PCR Detection System (Bio-Rad, Hercules, CA, USA). Each reaction volume of 25.0 μL contained 1.0 μL (0.4 μM) of each primer, 2 μL of diluted cDNA mix, 12.5 μL of 2 × TB Green Premix Ex TaqII (TaKaRa, Beijng, Japan), and 8.5 μL of RNA-free water. The experiment included three independent biological and technical replicates. The LightCycler Relative Quantification Software 4.05 was used to determine the relative abundance of each unigene. Correlation analysis was conducted using the Pearson correlation coefficient (PCC) to compare RNA-seq normalized counts with qPCR relative gene expression.

### 2.6. Statistical Analysis of Data

The metabolome determination, transcriptomic sequencing, proteomic analysis, and qRT-PCR analysis each underwent three biological replicates. Data were analyzed using Excel 2019 (Microsoft Inc., Redmond, WA, USA) and origin 2021. Heatmaps were generated using TBtools. Adobe Illustrator CS6 (Adobe Inc., San Jose, CA, USA) was used to assemble the figures and draw the flow diagram.

## 3. Results

### 3.1. Metabolomic Analysis of Lacquer Tree Under High-Temperature Stress

**Figure 1:**
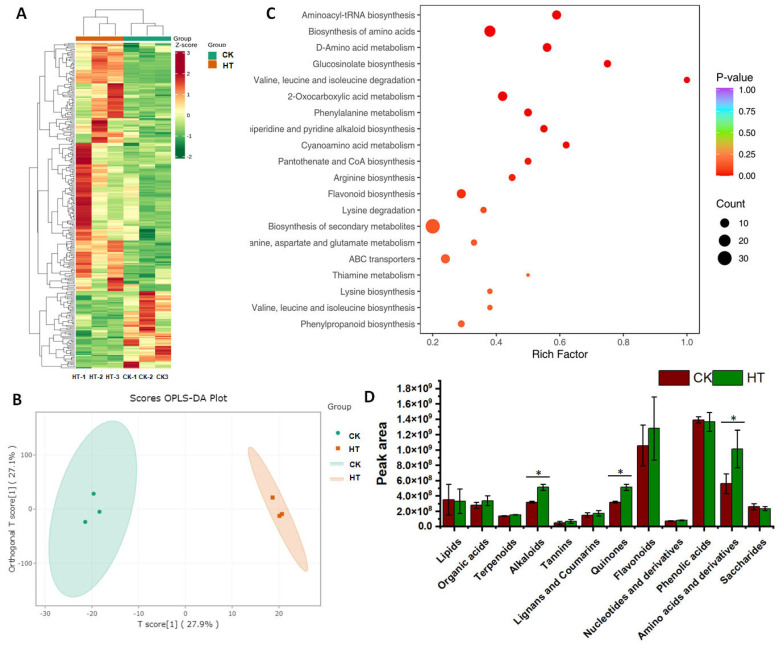
The heatmap of different expressed metabolites in high-temperature-treated samples and control (**A**), PCA analysis of the metabolites in high-temperature-treated samples and control (**B**), the KEGG pathway enrichment analysis of the differentially expressed metabolites (**C**), and total content of 12 categories in high-temperature-treated samples and control (**D**). *, <0.05.

A total of 1697 metabolites were analyzed from the two groups of samples, with 224 metabolites’ content upregulated in high-temperature-treated samples and 69 metabolites’ content downregulated in high-temperature-treated samples (Figure 1A). The principal component analysis showed that the metabolites’ content in T. vernicifluum under high temperature was profoundly changed (Figure 1B). The KEGG pathway enrichment analysis of the differentially expressed metabolites (DEMs) showed most of them enriched in the biosynthesis of secondary metabolites (Ko01110) and biosynthesis of amino acid (Ko01230) of metabolism pathways (Figure 1C). The total content of alkaloid amino acids, derivatives, and quinones was significantly upregulated in the high-temperature-treated samples (Figure 1D). However, there were no significant differences in the total content of lipids, organic acids, terpenoids, tannins, lignans and coumarins, flavonoids, nucleotides and derivatives, phenolic acids, and saccharides, although the content of some compounds within these categories showed significant changes such as Syringaresinol and Naringenin-7-O-(6″-malonyl)glucoside (Figure 1D and Supplementary Table S2).

### 3.2. Transcriptomic Analysis of Lacquer Tree under High-Temperature Stress

**Figure 2:**
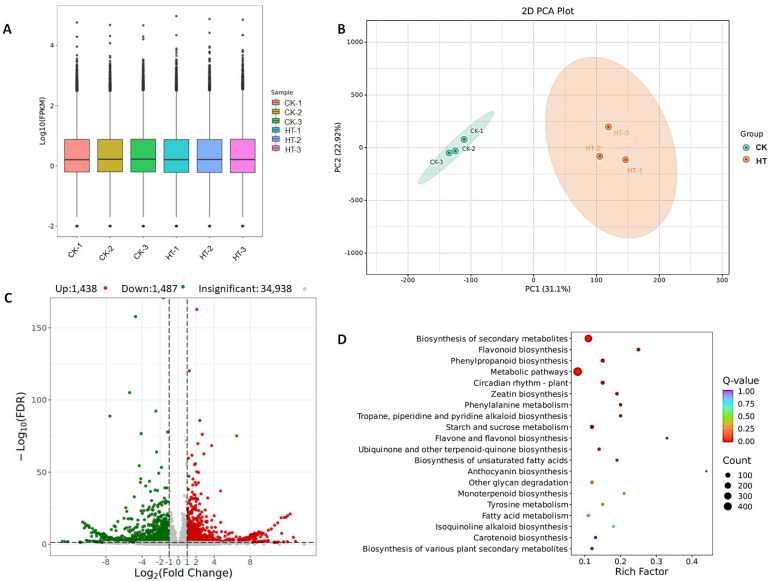
Boxplot of the gene expression in high-temperature-treated samples and control (**A**), PCA analysis of the expression of genes in high-temperature-treated samples and control (**B**), volcano plot of different expressed genes in high-temperature-treated samples and control (**C**), and the KEGG pathway enrichment analysis of the differentially expressed genes (**D**).

In the transcriptome analysis, the variation in gene expression levels within individual samples appears to be moderate, suggesting a balanced representation across the dataset (Figure 2A). Moreover, the overall gene expression profiles exhibit a notable degree of consistency across different samples, as depicted in Figure 2A. Principal component analysis further elucidated these findings, demonstrating that the gene expression patterns within the control group samples and the high-temperature treatment group samples are comparably homogeneous, while distinct differences are observed between the control and high-temperature-treated samples (Figure 2B). The differential expression analysis identified a total of 2925 unigenes showing significant changes, with 1438 unigenes upregulated and 1487 unigenes downregulated in response to the high-temperature treatment (Figure 2C). Enrichment analysis of the differentially expressed genes using the KEGG database revealed a predominant enrichment of genes involved in metabolic pathways and the biosynthesis of secondary metabolites (Figure 2D). This suggests that the high-temperature treatment has a notable impact on the expression of genes associated with metabolite synthesis and related pathways, underscoring the biological significance of the observed transcriptional changes.

### 3.3. Proteomic Analysis of Lacquer Tree under High-Temperature Stress

**Figure 3:**
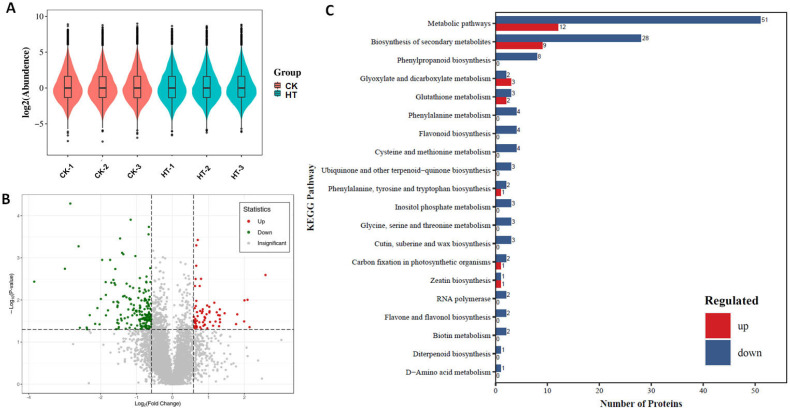
Violin plot of the protein expression in high-temperature-treated samples and control (**A**), volcano plot of different expressed proteins in high-temperature-treated samples and control (**B**), and the KEGG pathway enrichment analysis of the differentially accumulated proteins (**C**).

The 4D-DIA proteomics techniques were adeptly applied to scrutinize the alterations in protein expression patterns within lacquer trees subjected to high-temperature stress. Leveraging this methodology, an impressive total of 64,438 peptides were meticulously identified and assembled, culminating in the elucidation of 7920 distinct proteins. The remarkable coherence observed in the dispersion, distribution, and probability density of protein expression across all samples serves as a testament to the high quality and reliability of the proteomic dataset, as depicted in Figure 3A. Among these proteins, a subset of 245 exhibited differential expression in response to high-temperature treatment, as illustrated in Figure 3B. Specifically, 64 proteins were found to be upregulated, while 181 proteins were downregulated in samples subjected to elevated temperatures (Figure 3B). Delving deeper into the functional implications of these differentially accumulated proteins, KEGG enrichment analysis unveiled a predominant enrichment of proteins associated with metabolic pathways and the biosynthesis of secondary metabolites, mirroring the findings from the transcriptomic analysis (Figure 3C). This consistency between the proteomic and transcriptomic datasets underscores the robustness and reliability of the observed molecular responses to high-temperature stress. Such concordance between different omics approaches enhances our confidence in the biological relevance of the identified pathways and processes implicated in the adaptive responses of lacquer trees to thermal stress.

### 3.4. High Temperature Induced Degradation of ABA and Accumulation of Jasmonoyl-L-Isoleucine and Methylsalicylic Acid in Lacquer Tree

**Figure 4:**
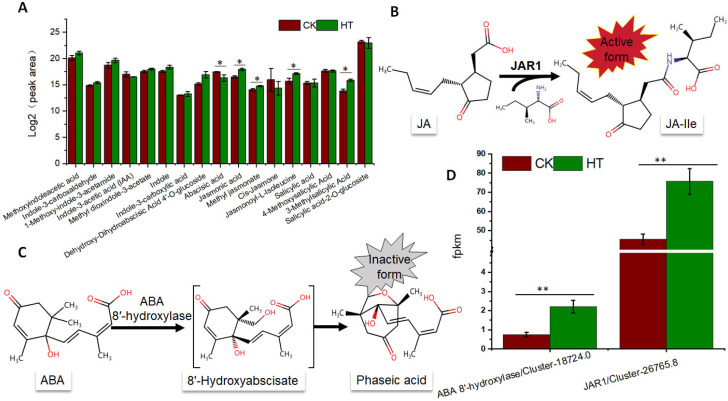
The content of identified plant hormones and derivatives in high-temperature-treated samples and control (**A**), the formation of JA-Ile catalyzed by JAR1 (**B**), the degradation of ABA catalyzed by ABA 8′-hydroxylase (**C**), and the expression of JAR1 and ABA 8′-hydroxylase encoding genes (**D**). *, <0.05; **, <0.01.

Environmental stressors commonly disrupt the delicate hormonal equilibrium within plants, compelling us to delve into an exhaustive analysis of plant hormone dynamics within lacquer trees subjected to high-temperature stress. Our investigation encompassed a comprehensive examination of pivotal hormones, such as indole-3-acetic acid (IAA), abscisic acid, jasmonic acid, salicylic acid, and their respective derivatives (Figure 4A). Despite the challenging conditions imposed by high temperatures, the levels of indole-3-acetic acid and its derivatives, including methoxyindoleacetic acid, indole-3-carboxaldehyde, 1-Methoxy-indole-3-acetamide, methyl dioxindole-3-acetate, indole, and indole-3-carboxylic acid, exhibited no significant fluctuations (Figure 4A). However, a conspicuous reduction in abscisic acid content was observed, concomitant with an elevation in dihydroabscisic acid 4′-O-glucoside, indicative of non-active derivatives of abscisic acid (Figure 4A). Furthermore, the levels of jasmonoyl-L-isoleucine, active forms of jasmonic acid, and 3-methylsalicylic acid showed a marked increase in lacquer trees under high-temperature stress. The conversion of jasmonic acid to jasmonoyl-L-isoleucine was facilitated by the enzyme JASMONATE RESISTANT1 (JAR1) (Figure 4B), while the inactivation of ABA was catalyzed by ABA 8′-hydroxylase (Figure 4C). Consequently, we scrutinized the expression patterns of genes encoding JAR1 and ABA 8′-hydroxylase. Remarkably, the expression levels of these genes exhibited a significant upregulation in response to the imposition of high temperatures (Figure 4D). This elucidates the pivotal roles played by JAR1 and ABA 8′-hydroxylase in modulating the hormonal responses of lacquer trees to environmental stresses, shedding light on the intricate regulatory mechanisms orchestrating their adaptation to adverse conditions.

### 3.5. High-Temperature Activation of Nitrogen Metabolism in Lacquer Trees

**Figure 5:**
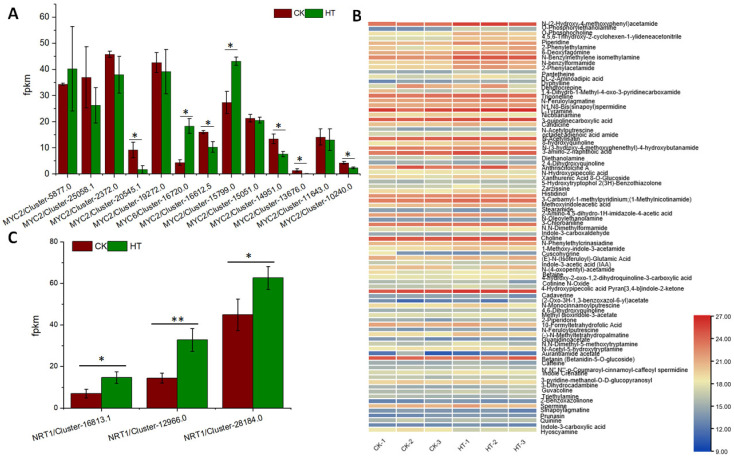
The expression of MYC transcription factor encoding genes in high-temperature-treated samples and control (**A**), the content heatmap of alkaloids in high-temperature-treated samples and control (**B**), the expression of NRT1 and NRT2 encoding genes (**C**). *, <0.05; **, <0.01.

The elevation in temperature triggered a notable accumulation of jasmonic acid (JA), jasmonoyl-L-isoleucine, and 3-methylsalicylic acid, potent stimulators of plant defense mechanisms against pests and diseases. Given the pivotal role of jasmonoyl-L-isoleucine in inducing the MYC transcription factor, we meticulously examined the expression profiles of MYC encoding genes in lacquer trees subjected to high-temperature stress. Intriguingly, our analysis revealed the significant induction of two MYC encoding genes in response to heightened temperatures within lacquer trees (Figure 5A), elucidating the intricate molecular responses underlying temperature-induced stress. In their quest to bolster resilience against pests and diseases, plants employ a myriad of defense strategies, including the synthesis of toxic compounds. Alkaloids, renowned for their ability to fend off pathogens and pests, were found to exhibit a significant increase in total content within lacquer trees under high-temperature stress conditions (Figure 1D). The heatmap representation of the alkaloid content in both control and high-temperature-treated samples further accentuated this surge (Figure 5B). As the alkaloid content surged, an ancillary response was observed in the activation of the nitrogen transport system. To elucidate this phenomenon, we delved into the expression patterns of NITRATE TRANSPORTER (NRT) encoding genes. Notably, the expression levels of three NITRATE TRANSPORTER encoding genes displayed a significant increase in lacquer trees subjected to high temperatures (Figure 5C), indicating a concerted effort to optimize nutrient uptake and allocation amidst challenging environmental conditions.

**Figure 6:**
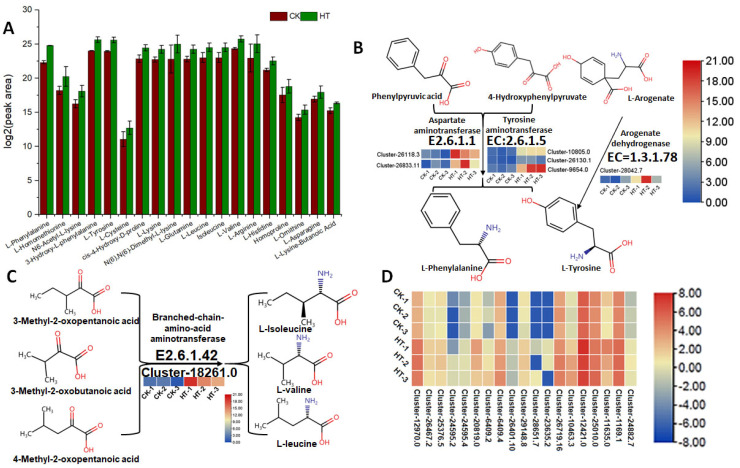
The content of identified amino acids and derivatives in high-temperature-treated samples and control (**A**), the expression of enzymes encoding genes in the formation of L-phenylalanine and L-tyrosine (**B**), the expression of enzyme encoding gene in the formation of leucine, isoleucine and valine (**C**), the expression heatmap of protease encoding genes in high-temperature-treated samples and control (**D**).

The total content of amino acids and derivatives notably increased in samples treated at high temperature. Specifically, the levels of L-phenylalanine, 3-hHydroxy-L-phenylalanine, L-tyrosine, L-valine, and L-lysine-butanoic acid showed significant enhancements (Figure 6A). In the transcriptome and proteome data, the expression of the enzymes or genes encoding enzymes involved in the synthesis of the phenylalanine or valine skeleton did not increase. However, the aminotransferase encoding genes responsible for the formation of phenylalanine, tyrosine, and valine increased (Figure 6B,C). We further analyzed enzymes related to protein degradation, and the results showed that the expression of 19 protease-encoding genes was upregulated under high temperature (Figure 6D).

### 3.6. Flavonoid Glycosides Increased in Lacquer Trees Under High Temperature

**Figure 7:**
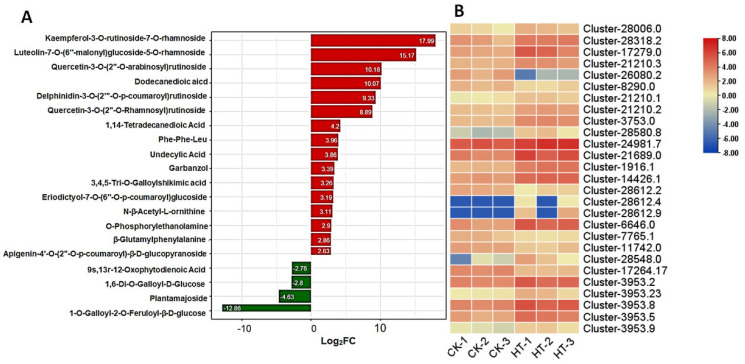
The 20 compounds exhibiting the most significant content changes following high-temperature treatment ((**A**), red: upregulated; green: down regulated), and the expression heatmap of protease encoding genes in high-temperature-treated samples and control (**B**).

Through our comprehensive metabolomic analysis, we meticulously examined alterations in the chemical composition of flavonoid glycosides. Notably, we observed a remarkable increase in the content of specific flavonoid glycosides, such as kaempferol-3-O-rutinoside-7-O-rhamnoside, luteolin-7-O-(6″-malonyl) glucoside-5-O-rhamnoside, and quercetin-3-O-(2″-O-arabinosyl) rutinoside, as illustrated in Figure 7A. These compounds exhibited the most substantial surge in concentration levels. In elucidating the biochemical pathways involved, we identified glycosyltransferases as pivotal catalysts responsible for the biosynthesis of flavonoid glycosides. Notably, our analysis revealed a significant upregulation of 27 genes encoding glycosyltransferases under conditions of high-temperature treatment, as depicted in Figure 7B. Furthermore, it is noteworthy that a subset of these genes exhibited concurrent upregulation at the protein level, further underscoring the importance of glycosyltransferases in mediating the observed metabolic responses to high temperature.

### 3.7. Analysis of the Regulatory Network of Lacquer Trees under High Temperature Using WPCNA and PPI

**Figure 8:**
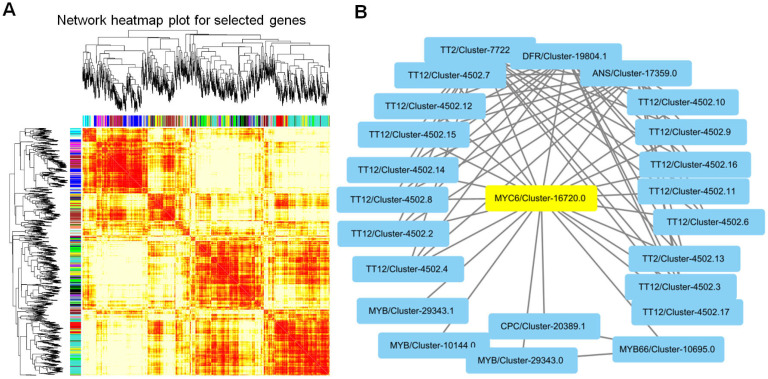
The hierarchical clustering heatmap of the module proteins (**A**), and the proteins interacted with MYC6 (**B**).

Weighted Protein Co-expression Network Analysis (WPCNA) was employed to investigate the associations among the expressed proteins. A hierarchical clustering heatmap displaying the module proteins is depicted in Figure 8A, revealing a total of 35 module network node proteins. Notably, the black module comprised genes linked to secondary metabolite biosynthesis, while the blue module encompassed genes associated with protein phosphorylation and signal transduction. Moreover, the orange, sky blue, yellow, and green-yellow modules housed the MYC component of the jasmonic acid signaling pathway, exhibiting robust correlations among themselves (Figure 8A). To delve deeper into the downstream effects of MYC6/Cluster-16720.0, protein–protein interaction (PPI) analysis was conducted (Figure 8B). Remarkably, several proteins, including the MBY transcription factor, DFR, ANS, and TT12, were identified as participants in flavonoid biosynthesis, particularly anthocyanins. These proteins were found to interact with MYC6, aligning with the observed upregulation of numerous flavonoids.

## 4. Discussion

A broad range of research focused on understanding how organisms adjust their metabolic processes in response to elevated temperatures [14]. This field of study involves investigating the changes in gene expression, protein abundance, and metabolite levels that occur when organisms are subjected to high-temperature conditions [15]. Various omics technologies, such as transcriptomics, proteomics, and metabolomics, are used to comprehensively analyze these molecular responses [16]. Key areas of investigation within this topic include identifying the specific metabolic pathways that are affected by heat stress, uncovering the regulatory mechanisms that govern metabolic reprogramming, and elucidating the physiological consequences of metabolic changes under high-temperature conditions [15]. High temperatures can trigger a restructuring and regulation of plant metabolic pathways [17], as observed in the significant changes in the content of 293 metabolites in lacquer trees subjected to high temperatures. This alteration in metabolic pathways often leads to changes in the synthesis and accumulation of secondary metabolites, such as phenolic compounds, alkaloids, and tannins. These changes are crucial for the plant’s physiological responses and defense mechanisms against stress [10]. Additionally, the biosynthesis of alkaloids, quinones, amino acids, and their derivatives is activated in lacquer trees under high-temperature conditions, as indicated in Figure 1D. This suggests a complex metabolic response of lacquer trees to high-temperature stress, involving various biochemical pathways to cope with the adverse environmental conditions. To explore the mechanism underlying the alteration in metabolite content induced by high temperature, we conducted transcriptomic and metabolomic analyses. The results revealed that both transcriptomic and proteomic analyses identified several differentially expressed genes involved in metabolic pathways. The expression of genes involved in secondary metabolite biosynthesis also significantly changed in Paspalum wettsteinii under high temperature [18]. This suggests a coordinated response at the transcriptional and protein levels to high-temperature stress, leading to alterations in metabolic pathways in plants.

Environmental stress is often transformed into plant hormonal responses [19]. High temperature also induces changes in plant hormones [20]. In our study, we observed a decrease in ABA content under high-temperature conditions (Figure 4A), accompanied by an upregulation in the expression of the ABA 8′-hydroxyabscisate encoding gene, which catalyzes the degradation of ABA (Figure 4D). When temperatures rise, the breakdown of ABA is triggered, altering the hormonal balance within the trees. However, the content of ABA was upregulated under high temperature in *Zea mays* [20]. This may be due to the species variations. In contrast to ABA, the levels of jasmonic acid and its active form, jasmonoyl-isoleucine, increased under high-temperature conditions (Figure 4A), accompanied by an upregulation in the expression of JAR1; encoding enzymes catalyzes the formation of jasmonoyl-isoleucine (Figure 4D). In maize seedlings, the content of jasmonic acid decreases under high-temperature stress [20]. Different plant species may exhibit varying hormone response patterns to high temperatures. JA and its derivatives serve as crucial signaling molecules in stress response pathways [21]. The elevated levels of JA and derivatives play pivotal roles in orchestrating various adaptive mechanisms aimed at mitigating the detrimental effects of high-temperature stress [21]. From bolstering defense responses to modulating growth and development, the dynamic interplay between ABA degradation and JA accumulation underscores the intricate regulatory mechanisms employed by lacquer trees to navigate environmental challenges posed by high temperature.

Nitrate is the preferred form of nitrogen for most plants and mediates ABA accumulation [22]. In turn, ABA induces the expression of root-type NRT2/NAR genes [23]. However, the breakdown of ABA resulted in the downregulation of NRT2 (Figure 5C). NRT1, which redistributes nitrate into developing tissues, was upregulated. The upregulation of NRT1 may be due to the acceleration of JAs signals. The interplay of the ethylene/jasmonic acid-NRT signaling module regulates the redistribution of nitrate and balances between growth promotion and environmental resilience [24]. Some MYC transcription factors, upregulated by JAs [25], were upregulated in lacquer trees under high temperature. MYC may regulate the expression of NRT1 and aminotransferases impacting nitrogen assimilation, alkaloids, and amino acid synthesis. JAs also activate nitrogen metabolism by inducing the expression of proteases [26]. Some proteases are upregulated in lacquer trees under high temperature (Figure 6D). This may be regulated by the increased JA signal induced by high temperature in lacquer trees.

The activation of the MYC-regulated flavonoid synthesis pathway represents a pivotal aspect of plant response to environmental stress [27]. Under high-temperature stress conditions, plants initiate a cascade of molecular events aimed at enhancing their resilience. In lacquer trees, high temperature led to an increase in the content of JAs and activated the jasmonic acid signaling pathway. In this signaling pathway, particularly through its derivative, jasmonoyl-isoleucine acts as a crucial mediator in this process [28]. Jasmonoyl-isoleucine activates MYC transcription factors, which subsequently regulate the expression of genes involved in flavonoid biosynthesis [29]. Flavonoids play multifaceted roles in plants, including antioxidant defense, UV protection, and defense against pathogens [30]. Their synthesis is intricately regulated to meet the demands of changing environmental conditions [31]. High temperatures trigger the expression of MYC transcription factors, which, in turn, upregulate the expression of MBY transcription factors and key enzymes in the flavonoid biosynthetic pathway [27]. These enzymes catalyze the conversion of precursor molecules into various flavonoid compounds, such as flavonols, flavones, and anthocyanins [32]. In lacquer trees, the upregulation of JAs in response to high temperature serves several purposes. Firstly, JAs activated flavonoid synthesis through MYC and MYB transcription factors. Flavonoids act as antioxidants, scavenging reactive oxygen species generated under stress conditions and preventing oxidative damage to cellular components [31]. Secondly, flavonoids contribute to the modulation of plant growth and development, serving as signaling molecules in various physiological processes [31]. Additionally, flavonoids play a crucial role in plant defense against biotic and abiotic stresses by inhibiting the growth of pathogens and pests and protecting plants from UV radiation [30]. Flavonoids protect against oxidative damage by scavenging reactive oxygen species (ROS), enhancing antioxidant enzyme activity (e.g., superoxide dismutase), chelating metal ions (like iron), regulating signaling pathways, and preventing lipid peroxidation, thereby promoting overall cellular health, as seen in their roles in plant stress tolerance and human health benefits [33]. Understanding the intricate interplay between high temperature, jasmonic acid signaling, and MYC-regulated flavonoid synthesis provides valuable insights into plant adaptation strategies and may inform the development of innovative approaches to enhance crop resilience and productivity in the face of climate-change-induced environmental fluctuations.

## 5. Conclusions

Lacquer trees are an important economic species that frequently face high-temperature stress during summer. Understanding how lacquer trees respond to high-temperature stress through multiple omics analyses is crucial for their cultivation and management. Under high-temperature treatment, the metabolite content of lacquer trees undergoes changes, with significant increases observed in alkaloids, quinones, amino acids, and their derivatives. The elevated levels of alkaloids, amino acids, and derivatives indicate active nitrogen metabolism. Further analysis reveals that high temperatures induce the degradation of abscisic acid in lacquer trees, leading to an increase in jasmonic acid (JA) content. JAs activate the MCY transcription factor, which further enhances the expression of NRT1, facilitating nitrogen transport within the lacquer tree. This activation also stimulates the expression of amino transferase and protease, promoting protein degradation and amino acid metabolism, thus activating nitrogen metabolism. Additionally, MCY activates the expression of glycosyltransferase, promoting the glycosylation modification of flavonoids, resulting in the greatest increase in flavonoid glycoside content under high-temperature treatment. By uncovering the metabolic changes and molecular mechanisms underlying the response to high temperatures, this research provides valuable insights for the cultivation and management of lacquer trees. The findings highlight the activation of nitrogen metabolism pathways, the role of JAs in mediating stress response, and the modulation of flavonoid glycosylation under high-temperature conditions. Understanding these mechanisms not only enhances our knowledge of plant stress responses but also offers potential strategies for improving lacquer tree resilience and productivity in the face of climate change. Overall, our study contributes significantly to both basic plant science and practical applications in agriculture and forestry.

## Data Availability

The data that support the findings of this study are available from the corresponding author upon reasonable request.

## Supplementary Materials

The following supporting information can be downloaded at: https://www.mdpi.com/article/10.3390/biology13110876/s1, Figure S1: High-temperature-treated seedlings and control seedlings of lacquer tree; Table S1: qPCR primers and gene coefficient of correlation between RNA-seq and gene expression; Table S2: The content of identified compounds.
