# Supplementary material for: Multiple Omics Analyses Reveal Activation of Nitrogen Metabolism and Flavonoid Glycosylation in Toxicodendron vernicifluum Under High Temperature
Source: Biology (Basel). 2024 Oct 28;13(11):876. doi: 10.3390/biology13110876 (PMC11591865; doi:10.3390/biology13110876)
Supplement: Supplementary file 1 [file biology-13-00876-s001.zip › Supplementary Table S1.pdf]

Table S1. qPCR primers and gene coefficient of correlation between RNA-seq and gene expression.

Gene ID	Forward / Reverse primer sequence	R^2 Correlation Coefficient	P value
Cluster-20545.1	CATTCTACATATTTATAATG/GCTGCAGAAGACGGTAAAAG	0.920	0.327
Cluster-16720.0	CTTTTATGGCAACATATTCC/GAAGATTTTGTTTGAAGTTC	0.922	0.231
Cluster-16612.5	GAGAAGCCAACAGCTTAGAG/CATTTGCTGGCGGGTCGACTG	0.902	0.238
Cluster-18724.0	CTCAGGAGGCTTGTTCAGAC/CTTCTGCTCTGCCTTTACAG	0.932	2.981
Cluster-26765.8	GTCCAAACAATTGCAGCATTC/GTATTGATTGTTAATAGGTG	0.891	0.242
Cluster-15799.0	CGAATCATGGCTTCTGATCC/CTAACCGAAGTGTTCCTCTC	0.895	0.923
Cluster-14951.0	CCAAGTTCTCACATTACAAC/GTAGAAGAGTTGGAATCCTG	0.933	0.285
Cluster-13676.0	CAACCACAACTAGTCCACAC/CTGATTCGGAGAACCTCTTG	0.874	1.231
Cluster-10240.0	CACACACGTAGAAGTACAAG/GTTTCTGTAGCTCCTCCTCC	0.914	0.367
Cluster-16813.1	CTCCATTTGCAAAGCTTTAG/CTTTTTGGACCATTTTTGCG	0.925	0.425
Cluster-12966.0	CCGAAAACTCGGCTTGGCTC/GATTTATAGATTCTTGTTGC	0.912	0.325
Cluster-28184.0	CTTAATGCAGCTAGAAGTCC/CAGTAGAACAAGCTTCCACC	0.911	0.453
Cluster-26118.3	CAACACGACTCCGTTTTCTC/CTGATTGTGCATCTGCATCC	0.889	1.276
Cluster-26833.11	CCTAATGCAGTTAGGCAAGC/CATTTGTCCACTGACTGTAC	0.914	1.341
Cluster-10805.0	GATGATAACTTGTACGGAAG/CAATTTCCAGATTCCAATCC	0.927	1.027
Cluster-26130.1	GCTTTGTTGATGTCATCGTG/CTTCTCTCCCTCATCGTCAG	0.931	0.315
Cluster-9654.0	CTGCCTAACGCTTCCTCGAG/GTTATCTCTTGTGAGATTGA	0.951	0.245
Cluster-28042.7	GATTCTGCAGCTCTTGCATG/CAACTTCTTCTTCTTCCTCC	0.942	0.423
Cluster-24882.7	GGCTCGCTTGTTATTACATG/GACCTGATTGATGTCTGATG	0.941	0.321
Actin/ Cluster6035.0	CTACTCAAATGAAGCGCATG/GTAGAGTATGCTGCTCTATC	-	-
