# Supplementary figures and images for: Multiple Omics Analyses Reveal Activation of Nitrogen Metabolism and Flavonoid Glycosylation in Toxicodendron vernicifluum Under High Temperature
Source: Biology (Basel). 2024 Oct 28;13(11):876. doi: 10.3390/biology13110876 (PMC11591865; doi:10.3390/biology13110876)

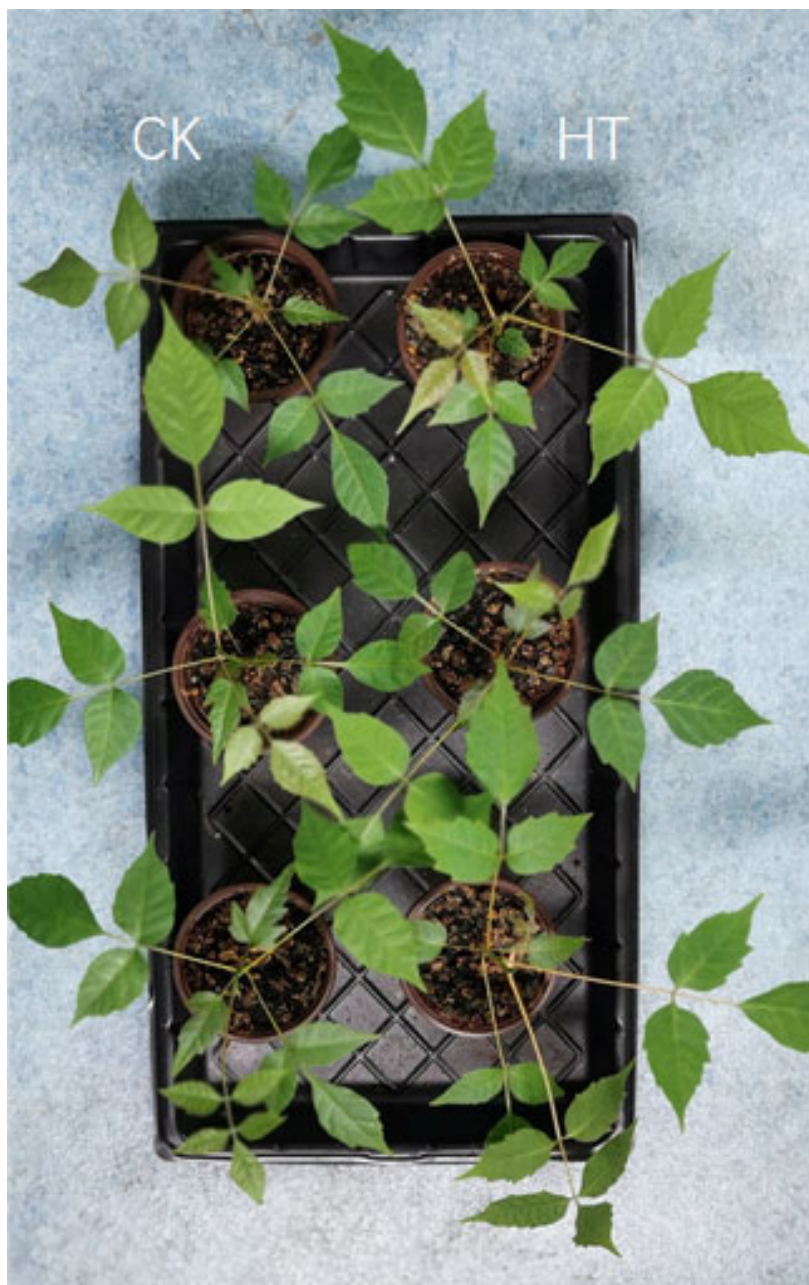

**Figure S1.** High-temperature treated seedlings (HT) and control seedlings (CK) of lacquer tree.

Supplement: Supplementary file 1 [file biology-13-00876-s001.zip › Supplementary Figure S1.pdf]
